# Getting with the times: a narrative review of the literature on group decision making in virtual environments and implications for promotions committees

**DOI:** 10.1007/s40037-018-0434-9

**Published:** 2018-05-24

**Authors:** Anita Acai, Ranil R. Sonnadara, Thomas A. O’Neill

**Affiliations:** 1 Department of Psychology, Neuroscience & Behaviour, McMaster University, Hamilton, Ontario Canada; 2 Office of Education Science, Department of Surgery, McMaster University, Hamilton, Ontario Canada; 3 Department of Surgery, University of Toronto, Toronto, Ontario Canada; 4 Department of Psychology, University of Calgary, Calgary, Alberta Canada

**Keywords:** Promotions committees, Competence committees, Group decision making, Trainee promotion, Technology, Virtual meetings

## Abstract

**Introduction:**

Concerns around the time and administrative burden of trainee promotion processes have been reported, making virtual meetings an attractive option for promotions committees in undergraduate and postgraduate medicine. However, whether such meetings can uphold the integrity of decision-making processes has yet to be explored. This narrative review aimed to summarize the literature on decision making in virtual teams, discuss ways to improve the effectiveness of virtual teams, and explore their implications for practice.

**Methods:**

In August 2017, the Web of Science platform was searched with the terms ‘decision making’ AND ‘virtual teams’ for articles published within the last 20 years. The search yielded 336 articles, which was narrowed down to a final set of 188 articles. A subset of these, subjectively deemed to be of high-quality and relevant to the work of promotions committees, was included in this review.

**Results:**

Virtual team functioning was explored with respect to team composition and development, idea generation and selection, group memory, and communication. While virtual teams were found to potentially offer a number of key benefits over face-to-face meetings including convenience and scheduling flexibility, inclusion of members at remote sites, and enhanced idea generation and external storage, these benefits must be carefully weighed against potential challenges involving planning and coordination, integration of perspectives, and relational conflict among members, all of which can potentially reduce decision-making quality.

**Discussion:**

Avenues to address these issues and maximize the outcomes of virtual promotions meetings are offered in light of the evidence.

## What this paper adds

The use of virtual meetings by promotions committees, both undergraduate and postgraduate (e.g., competence committees, which are tasked with making resident promotion decisions in competency-based residency training programs), is nearly inevitable as committees struggle to accommodate challenges with respect to scheduling and logistics. However, best practices for upholding the integrity of decision-making processes related to trainee promotion requires further consideration. This narrative review lays a foundation for discussion and scholarship relating to this topic by integrating findings from disciplines such as psychology and organizational behaviour, where virtual meetings have been the subject of extensive research.

## Introduction

In the wake of major changes in health professions education (HPE), including a recent global shift towards competency-based medical education (CBME), educators report an increased time and administrative burden for assessment and assessment-related activities [1]. Not only does CBME emphasize assessment frequency and quality of feedback, it also requires new ways of synthesizing assessment data to make promotions decisions [2]. Competence committees (aka clinical competency committees in the United States (US)) play a central role in the assessment of trainees’ progressive attainment of competence [3]. Competence committees are comprised of educators, and in some cases senior trainees and community members, who consider data from multiple sources to evaluate trainees’ progress and assess readiness to move to the next stage of training [3]. In Canada, the US, and the United Kingdom, accreditors have mandated implementation of competence committees [3–5].

Researchers have reported concerns related to the time and administrative burden of CBME in relation to competence committees [6]. For example, time pressures have been proposed as threats to the quality of competence committee decisions [7]. To meet these challenges virtual meetings, an attractive and popular alternative to face-to-face meetings, have been proposed as a potential mitigation strategy [8, 9].

Munro and Swartzman define virtual meetings as those involving ‘participants who may, or may not, be part of a permanent team and who interact with each other non-simultaneously using shared … data. They may operate at a local or a national level’ [9]. They purposefully do not specify a virtual communication modality (e.g., video conference), as these may vary by team. Although virtual meetings could potentially address some of these concerns there is uncertainty as to whether they would maintain the integrity of decision-making processes [10]. Clearly, this is critical to ensuring the reliability, validity, fairness, and defensiveness (including legal) of trainee assessment and promotion processes. Thus, a more thorough understanding of the benefits and challenges of decision making in virtual environments may help inform the structure and processes used by competence committees as they are implemented more broadly.

This narrative review aims to summarize the existing literature on virtual meetings, discuss potential ways they can impact decision making, and explore implications for promotions committees. Although we conceived of this review while studying competence committees in graduate medical education, we feel that it also has implications for other promotions committees, such as those in undergraduate medical education [11].

## Methods

We have conducted a narrative review [12] to synthesize the literature on virtual teams and explore its potential application to the work of promotions committees. We used Green et al.’s approach to narrative review writing, which emphasizes the importance of reporting sources, search terms, inclusion and exclusion criteria, and limitations in preparing high-quality narrative reviews [13].

In August 2017, we searched Web of Science (WoS) using ‘decision making’ AND ‘virtual teams.’ Our search was limited to peer-reviewed articles published in English between 1997–2017. This search yielded 336 articles, which were independently reviewed by the primary author (AA) to exclude those irrelevant to group decision making in virtual meetings. AA also manually searched *Perspectives on Medical Education *and the *Journal of Graduate Medical Education* between 1997 to 2017, as they were excluded in the WoS search. We also searched the reference lists of primary papers and added relevant articles not retrieved in our search. The final set included 188 articles. Using our subjective judgement as HPE (AA, RS) and virtual teams (TO) researchers, we selected a subset deemed of high-quality and relevant to promotions committees. For example, if a particular finding was determined using both a well-controlled experiment and a case study conducted in a local context, we cited the former. Moreover, we excluded articles we felt would be irrelevant to promotions committees (e.g., globalization and virtual team decision making). We selected this methodology as our aim was not to exhaustively review the characteristics of virtual teams, but rather to explore possible extensions of this literature to trainee promotion processes.

## Results

Based on our literature review, we identified several themes including team composition and development, idea generation and selection, group memory, and communication. These themes encompass various aspects of the virtual teams literature and overlap with factors thought to influence competence committee decision making, such as member characteristics, group size, group understanding of its work, group leader role, information-sharing procedures, and effects of time pressure [7].

### Team composition and development

Virtual team composition is a significant predictor of decision making effectiveness. Virtual teams with 13 or more members tend to exhibit the poorest outcomes; however, ‘social loafing’, people’s tendency to exert less effort in group work because they feel less responsible for the team’s output, has been observed in teams with as few as four members [14]. This is especially true when relying heavily upon asynchronous communication modalities (e.g., email or discussion boards) as the immediacy of feedback is generally quite low, making it much easier for members to disengage [15]. As in face-to-face communication, it is helpful to keep virtual teams small, including only the members necessary to complete tasks effectively [16]. Additionally, individual team member characteristics influence team performance [16]. For example, virtual teams with members less comfortable using technology tend to have lower participation levels, lower-quality contributions, and poorer member satisfaction than teams with members who are technologically savvy [17].

Virtual teams permit diverse membership with respect to gender, age, and race, as well as less visible traits such as knowledge, skills, and organizational tenure [8]. This is an area where there may be synergy between the factors that lead to optimal small group decision making and the benefits afforded by virtual teams, since competence committees with heterogeneous group membership are thought to perform better than those with homogenous group membership [7]. Despite benefits of diversity, challenges involving trust development, team cohesion, fault lines (subgroups), and integrating divergent perspectives may be exacerbated in diverse virtual teams [18].

### Idea generation and selection

Drawing on social decision scheme theory, [19] which describes the rules groups use to transition from individual preferences to collective decisions, Hauer et al. [7] emphasizes the importance of competence committees sharing information in their work. To this end, virtual teams generate more ideas using virtual media such as chat, instant messenger, and email [20]. This is due to less production blocking, which allows group members to generate information simultaneously without waiting for others to respond [21]. Team members may also be more likely share ideas virtually because it may feel less personal than in-person meetings [22]. However, generating more ideas does not necessarily mean making better decisions. In fact, meta-analyses indicate that virtual teams tend to underperform relative to face-to-face teams both in general, [23] and specifically with respect to decision making [24].

Poor decision making of virtual teams may be explained by ‘integrative complexity,’ which proposes that decision making consists of two opposing processes: differentiation and integration [25]. The paradox is that when a group excels at differentiation (i.e., idea generation), the group will have increased difficulty with the integration of perspectives. Moreover, when virtual team members receive information that challenges their pre-discussion preferences, it is processed in a similar way to irrelevant information [26]. Information, however, that supports an individual’s pre-discussion preference is processed more thoroughly. Thus, while virtual teams may have a larger selection of ideas to draw upon and are more likely to share minority opinions (i.e., successful conveyance), they are likely to encounter significant challenges with the convergence of perspectives [20], thereby leading to potentially worse decisions than face-to-face teams.

### Group memory and information processing

Chahine et al. [27] suggested that when group decision making is viewed from a constructivist paradigm, the focus shifts to how ‘group members collectively understand the purpose, implications, and potential alternatives of their group decision.’ Central to this perspective are shared mental models, which are essential for competence committees to ‘interpret information consistently, explain findings, and determine actions appropriately for their charge’ [7]. A related (and likely overlapping [28]) idea is that of a transactive memory system, which is a conceptual mechanism that includes not only individual team members’ knowledge, but also metamemory regarding different areas in which team members are knowledgeable [29, 30]. Substantial evidence suggests that virtual teams may find it harder to develop shared mental models and transactive memory systems due to physical distance, a lack of a collaborative history, and the high diversity of knowledge and expertise in virtual teams [31, 32]. Recall that virtual teams often generate more ideas than face-to-face teams, but integrating these ideas can often be more difficult and time-consuming [8].

Social factors can also impact virtual teams’ information processing and are particularly relevant to social influence orientations to group decision making, which ‘focus on how individuals within a group can be influenced and how perspectives may change based on social pressures’ [27]. Virtual teams may experience difficulties with planning and coordination, [33] which may manifest as less clarity around the roles, expertise, and thought processes of other members, and a poorer understanding of the task [8, 34]. Virtual team members may also find it challenging to build mutual trust because they may not have access to the social cues afforded by face-to-face communication [35]. This can lead to lower cohesion and greater prevalence of relationship conflict, [36] which has been linked to poorer team outcomes than task-related conflict [37]. It may also exacerbate pre-existing challenges in small group decision making such as groupthink, the tendency of group members to unquestioningly follow the group’s prevailing opinion in order to minimize conflict and maintain harmony [7]. Notably, however, these detrimental effects can be mitigated by effective leadership, [38] effective facilitation, and teaching teams to use more effective conflict management strategies [39].

### Communication and information processing

As argued by Hemmer and Kelly, [40] ‘conversation itself holds the key to understanding [competence committee] decisions.’ Thus, it is crucial to consider how virtual environments impact communication processes. Like group memory, communication directly influences the quality of information processing in virtual teams. The most important link between the two appears to be a team’s ability to evaluate the negative consequences of solutions, [41] which can be facilitated by constructive controversy through devil’s advocacy or dialectical inquiry [42]. These strategies are also effective antidotes to groupthink, and tend to work particularly well in virtual teams with strong fault lines or decision-making situations with many possible solutions or no obvious evaluation criteria [41]. This perspective is supported by Swaab et al.’s [43] communication orientation model, which indicates that a cooperative orientation overcomes barriers to information exchange by facilitating team trust, rapport, and identity.

Group memory and communication are also highly related because communication is the process by which teams evoke ideas from long-term memory and bring them to working memory for use in decision making [8]. Both implicit and explicit knowledge are important in this regard [44]. Implicit knowledge is best transmitted in direct team member interactions that incorporate nonverbal cues and, as a result, is more difficult to evoke in virtual team settings [44]. However, since communication technologies make it possible to create an ‘external memory’ of conversations, it is possible to mitigate this limitation by making certain implicit information—for example, rules related to coordination between team members—more explicit [8].

### Improving virtual team performance

We classified strategies to improve the effectiveness of virtual teams into four areas. First, the frequency and timing with which a team relies upon virtual communication appears to have an effect on overall performance. Teams that use face-to-face communication combined with virtual communication appear to be more successful than those reliant on virtual communication [45]. Many experts agree that the most critical window for face-to-face communication is upon initial formation of the team, as this facilitates development of trust and shared understanding of group tasks [45–47].

Second, communication modalities employed by the group should align with the group tasks based on a matching of communication features and task interaction requirements [48]. Maruping and Agarwal [15] provide a useful guide to matching various communication modalities with needs such as immediacy of feedback, symbol variety, parallelism, rehearsability, and reprocessability (see original paper for definitions). Their model is based on media synchronicity theory, which postulates that all communication can be broken down into two key processes: conveyance (the exchange of information and deliberation of its meaning) and convergence (the development of shared meaning of information) [20]. According to this theory, convergence processes are best supported by communication environments that enable high immediacy of feedback and low parallelism, such as face-to-face, while environments that enable low immediacy of feedback and high parallelism, such as email, are optimal for conveyance processes [20].

Training and interventions focused on augmenting group processes may facilitate virtual team effectiveness. Examples include training teams to manage conflict more effectively [39, 49–52]; introducing greater structure into the virtual team environment by adopting frameworks to help guide the decision-making process [53, 54]; engaging in group exercises to develop trust and communication skills [55]; and giving and receiving regular feedback about team performance [56].

Finally, effective leadership can impact factors that influence decision-making processes and tie together the preceding three groups of strategies [57–59]. Effective virtual team leaders should be proficient technology users and effective in helping team members monitor and manage their performance. Shared leadership, which occurs through the interrelation of individual and team actions, [60] supports performance outcomes in team-based environments regardless of the extent of virtuality [61–64].

## Discussion

This review considered the literature on decision making in virtual teams, exploring their functionality with respect to four key themes. Our findings suggest that while virtual teams potentially offer benefits, the benefits must be weighed against challenges involving planning and coordination, integration of perspectives, and relational conflict among members. What is promising, however, are the many strategies that promotions committees can use to facilitate good decision making in virtual environments. We next highlight key implications for promotions committees. A summary of the benefits and challenges of decision making in virtual teams and their implications for promotions committees is provided in Tab. 1.

**Table 1:** Summary of the benefits and challenges of decision making in virtual teams and their implications for promotions committees

Theme	Applicable domains of Hauer et al.’s [7] framework	Benefits and challenges of virtual teams	Implications for promotions committees
Team composition and development	– Group leader role – Group size – Member characteristics – See notes^a^	Benefits	– Ensure that members have access to, and are proficient in, the use of virtual communication technologies – Ensure that the committee has an effective chair who can help members establish clear goals, structures, and norms – Encourage regular, face-to-face meetings, especially when the committee is first formed – Spend the first meeting or two establishing clear guidelines for how meetings, both face-to-face and virtual, will be conducted, and specify the preparation and attendance expectations – Review the committee’s performance on an ongoing basis
		– Convenience and scheduling flexibility to include ideal team members	
		– Inclusion of members at remote sites; may help enhance membership diversity and access to knowledge and skills	
		Challenges	
		– Require members to have technological proficiency	
		– Member disengagement is more likely, particularly with larger virtual teams	
		– May exacerbate challenges involving trust development, team cohesion, faultlines (subgroups), and integrating divergent perspectives	
Idea generation and selection	– Effects of time pressure – Group leader role	Benefits	– Use a mix of meeting types—e.g., face-to-face when making high-stakes promotion decisions and virtual for communication about lower-stakes issues or administrative tasks
		– May generate more ideas due to less production blocking	
		– Members may be more likely to share minority opinions	
		Challenges	
		– May experience greater difficulty integrating perspectives	
		– As a result, can take longer to come to a high-quality decision than face-to-face teams	
Group memory and information processing	– Information-sharing procedures – Group leader role – Group understanding of its work	Benefits	– Create an easily accessible and up-to-date listing of committee members, their background and area(s) of expertise, current role(s), contact information, etc. This will help ensure that members are aware of who is currently on the committee, what skills they bring, and how best to contact them – Use technologies that allow for the ‘external storage’ of information, such as the contact list above. Virtual communication platforms such as Basecamp, Microsoft Teams, Slack, and Trello allow a record of files and interactions to be captured. Emails also allow external storage to some extent, but can be inefficient to manage, locate, and act on – In considering external storage solutions, ensure that institutional guidelines on data security and storage are adhered to. Information shared among promotions committee members is often sensitive and includes confidential information requiring secure storage and transmission
		– Diverse membership can allow for different kinds of knowledge across members; facilitates individual members’ learning and more efficient division of labour	
		– Allow for ‘external storage’ in the form of a communication record	
		Challenges	
		– May find it harder to develop shared mental models and knowledge of the different areas in which team members are knowledgeable	
		– May experiences challenges with planning and coordination	
		– More difficult to establish trust; can lead to poorer team cohesion and more relationship conflict	
		– May exacerbate pre-existing challenges such as groupthink	
Communication and information processing	– Information-sharing procedures – Group leader role – Group understanding of its work	Benefits	– Ensure that the communication modality being used is in alignment with the tasks that need to be accomplished and the overall needs of the team [15] – Be aware that neglecting face-to-face meetings can result in higher levels of intratream conflict and misunderstanding. Manage conflict pre-emptively by developing and maintaining trust and interpersonal relationships
		– Offer a range of communication modalities that can help members accomplish different tasks more efficiently	
		Challenges	
		– Greater potential for misunderstandings and team conflict	
		– May exacerbate pre-existing challenges such as groupthink	

^a^Although not included in the original framework by Hauer et al. [7], it may also be relevant to consider team development stages such as those proposed by Tuckman [65]: forming, storming, norming, performing, and adjourning

First, having an effective facilitator (i.e., chair) to help teams establish clear goals, structures, and norms is crucial to mitigate any challenges across the four identified themes. The chair attends to these issues throughout the committee’s development (e.g., Tuckman’s [65] forming, storming, norming, performing, and adjourning stages, which describe how teams begin as a group of individuals and, through a developmental process, can become a cohesive team before eventually disbanding). Additionally, the chair should utilize a structured meeting approach by dedicating the first few meetings to establishing clear guidelines for how meetings, both face-to-face and virtual, will be conducted, and specifying the preparation and attendance expectations. As part of this process, the committee should establish where documents will be stored, how to access them, and how to modify them so that there is only one current version—something that is often overlooked and can heavily tax coordination processes. The chair should also facilitate the monitoring and review of the committee’s performance informally through regular check-ins or formally, such as through an anonymous member survey annually or semi-annually. In Tab. 2, we provide effective facilitation strategies for virtual promotions committee meetings (although many also apply to face-to-face meetings) [66, 67].

**Table 2:** Effective facilitation strategies for virtual promotions committee meetings

When	Strategies
Before the start of the meeting	Familiarize yourself with the members who will be in attendance and their roles. Ensure that a virtual meeting is appropriate considering the committee’s membership and the task at hand
	Agree on the most appropriate technology platform upon scheduling the meeting (it is best to use hardware/software that both you and your members have used before or are familiar with). Test the technology in a ‘dry run’ prior to the meeting, ideally in the same room and with the same hardware/software
	Have a ‘Plan B’ for what you will do if the technology does not work—e.g., Is there a secondary platform that can be used? What steps will be taken to troubleshoot before moving on to this plan? Share this plan with members using a different modality (e.g., on paper or by email) so that they can still access the information if the primary method of communication fails
	Develop and circulate a clear agenda and meeting materials, adhering to institutional guidelines on data security and storage as appropriate. Seek members’ input regarding any additions or clarifications to the agenda
	Ensure that members have the information they need to connect virtually—e.g., instructions for downloading and installing appropriate software, number for calling in, etc
At the start of the meeting	Arrive at least 15 min early in order to help set up the meeting and ensure all technology is working. Encourage members connecting virtually to log in early so that the meeting start time is not delayed if the technology does not work right away
	Welcome members and ensure that introductions are done. Establish ground rules for the meeting—e.g., you might ask members to say their name each time they speak or use the mute button to eliminate background noises
	Briefly review the goals and expected structure of the meeting
During the meeting	Ensure that the meeting starts and ends on time and stays on track
	Ensure that there is turn-taking and roughly equitable contributions to the discussion, particularly from those connected virtually. It may be helpful to deliberately and explicitly check in with members who have not been saying very much
	Listen to, clarify, and periodically summarize information that has been discussed. If necessary, ask questions to clarify comments that may be unclear
	Address members who are not prepared or whose comments are inappropriate. Intervene early using appropriate strategies if conflict or negative emotions arise
	Ensure notes are taken so that actions and rationales are clear
	Maintain a positive and cooperative attitude that encourages and supports member participation and builds rapport and relationships
At the end of the meeting	Summarize the decisions made and ensure clarity on next steps, including who will be responsible for carrying out any action items
	Take stock by asking members to briefly reflect on what went well and what they would like to change for next time—e.g., by using the prompts ‘Stop,’ ‘Start,’ and ‘Continue.’ Ensure that any changes are implemented in time for the next meeting
	Follow-up with any members who may have ‘dropped out’ of the meeting due to technical difficulties to gather any contributions that may have been missed

*Sources: *Mittleman et al. [66]; Clawson et al. [67]

Committees need to establish clear guidelines on technology use. Not all tasks are optimal for virtual environments. For example, evidence suggests that the quality of decisions made virtually is generally poorer than those made face-to-face. Thus, we recommend that promotions committees avoid high-stakes promotions decisions virtually unless necessary (e.g., members located remotely with no opportunity to meet at a central location). However, virtual meetings may be excellent for communication about lower-stakes issues, or the coordination and completion of administrative tasks. Thus, a mix of meeting types is a compromise to address scheduling concerns and accommodate remote members while still maintaining the integrity of the decision-making process.

Timing of virtual meetings and the communication modality chosen are important considerations for virtual meetings. We recommend promotions committees still engage in face-to-face meetings, particularly when the committee is first formed so that members get to know one another and develop a sense of trust and rapport. Virtual meetings may be introduced later for scheduling flexibility or accommodating remote members. Neglecting face-to-face meetings can result in higher levels of intrateam conflict and misunderstanding; thus, promotions committees should manage conflict pre-emptively by developing and maintaining trust and interpersonal relationships. Swaab et al.’s communication orientation model [43] has a number of related important implications, suggesting that richer media and face-to-face meetings help members develop the rapport and trust needed to integrate information effectively, as does setting aside time for team members to get to know each other and their environments on a more personal level. Additionally, promotions committees may find it useful to use a framework such as Maruping and Agarwal’s [15] to align tasks with the most appropriate communication modality. Finally, having clear operating procedures that clarify members’ roles and responsibilities, and focusing on conflict management during team training and ongoing professional development (e.g., if conflict does arise, intervene early using appropriate conflict resolution strategies [39, 49–52]).

Finally, virtual promotions committees must consider data security and storage. Since promotions committees review confidential trainee performance data, meetings in a virtual environment may introduce data sharing challenges. Thus, promotions committees should ensure adherence with institutional guidelines on data security and storage. Dashboards, [68] which provide an electronic means of aggregating and summarizing quantitative and qualitative performance data, may offer a solution.

## Limitations and future directions

Currently the literature on virtual teams in promotion processes is limited; thus, we gleaned potential implications from fields such as psychology and organizational behaviour. While this approach allowed us to learn from the best practices of other disciplines, there could be factors unique to medical promotions committees that limit the generalizability of findings from other domains. Our search was limited to WoS, which means we may have inadvertently excluded articles not in this database. Nevertheless, this review identifies a need for future research. For example, researchers might question how promotions committees identify as a team or group, and how this informs their relationships and decision-making processes (e.g., collaboratively vs. autocratically). Future researchers may also investigate effects of virtual communication on decision-making processes in undergraduate and graduate promotion processes specifically, as well as the factors that may influence these processes to uncover the benefits, challenges, and best practices of adopting technology in this context.

## Conclusion

Virtual meetings are ubiquitous in many organizations and healthcare contexts. Leveraging literature from psychology and organizational behaviour can inform a better understanding of the benefits and challenges of virtual decision making.

## References

[1] Caverzagie KJ, Nousiainen MT, Ferguson PC (2017). Overarching challenges to the implementation of competency-based medical education. Med Teach.

[2] Holmboe ES, Sherbino J, Long DM, Swing SR, Frank JR, International CBME Collaborators (2010). The role of assessment in competency-based medical education. Med Teach.

[3] Andolsek K, Padmore J, Hauer KE, Holmboe E. Clinical competency committees: a guidebook for programs 2015 Available from: https://www.acgme.org/Portals/0/ACGMEClinicalCompetencyCommitteeGuidebook.pdf. Accessed January 2, 2018.

[4] Royal College of Physicians and Surgeons of Canada. Competence committees n.d. Available from: http://www.royalcollege.ca/rcsite/cbd/assessment/competence-committees-e. Accessed January 2, 2018.

[5] UK Foundation Programme (2017). Foundation annual review of competence progression (ARCP).

[6] Donato AA, Alweis R, Wenderoth S (2016). Design of a clinical competency committee to maximize formative feedback. J Community Hosp Intern Med Perspect.

[7] Hauer KE, ten Cate O, Boscardin CK (2016). Ensuring resident competence: a narrative review of the literature on group decision making to inform the work of clinical competency committees. J Grad Med Educ.

[8] Curşeu PL, Schalk R, Wessel I (2008). How do virtual teams process information? A literature review and implications for management. J Manage. Psychol..

[9] Munro AJ, Swartzman S (2013). What is a virtual multidisciplinary team (vMDT)?. Br J Cancer.

[10] Acai A, Cupido N, Weavers A, Sonnadara RR. Ready or not, here they come: early perceptions and experiences of competence committee implementation at a Canadian postgraduate medical training centre. In press 2018.

[11] Green EP, Gruppuso PA (2017). Justice and care: decision making by medical school student promotions committees. Med Educ.

[12] Grant MJ, Booth A (2009). A typology of reviews: an analysis of 14 review types and associated methodologies. Health Info Libr J.

[13] Green BN, Johnson CD, Adams A (2006). Writing narrative literature reviews for peer-reviewed journals: secrets of the trade. J Chiropr Med.

[14] Lepsinger R, DeRosa D (2010). Virtual team success: a practical guide for working and leading from a distance.

[15] Maruping LA, Agarwal R (2004). Managing team interpersonal processes through technology: a task-technology fit perspective. J Appl Psychol.

[16] Hackman JR (2002). Leading teams: setting the stage for great performances.

[17] Fuller RM, Vician CM, Brown SA (2016). Longitudinal effects of computer-mediated communication anxiety on interaction in virtual teams. IEEE Pro Commun..

[18] Garrison G, Wakefield RL, Xu XB, Kim SH (2010). Globally distributed teams: the effect of diversity on trust, cohesion and individual performance. Data Base Adv Inf Syst.

[19] Stasser G (1999). A primer of social decision scheme theory: models of group influence, competitive model-testing, and prospective modeling. Organ Behav Hum Decis Process.

[20] Dennis AR, Fuller RM, Valacich JS (2008). Media, tasks, and communication processes: a theory of media synchronicity. Manag Inf Syst Q.

[21] Dennis AR, Wixom BH, Vanderberg RJ (2001). Understanding fit and appropriation effects in group support systems via meta-analysis. Manag Inf Syst Q.

[22] Alnuaimi OA, Robert LP, Maruping LM (2010). Team size, dispersion, and social loafing in technology-supported teams: a perspective on the theory of moral disengagement. J Manag Inf Syst.

[23] Baltes BB, Dickson MW, Sherman MP, Bauer CC, LaGanke JS (2002). Computer-mediated communication and group decision making: a meta-analysis. Organ Behav Hum Decis Process.

[24] Lu L, Yuan YC, McLeod PL (2012). Twenty-five years of hidden profiles in group decision making: a meta-analysis. Pers Soc Psychol Rev.

[25] Chidambaram L, Tung LL (2005). Is out of sight, out of mind? An empirical study of social loafing in technology-supported groups. Inf. Syst. Res..

[26] Minas RK, Potter RF, Denis AR, Bartelt V, Bae S (2014). Putting on the thinking cap: using NeuroIS to understand information processing biases in virtual teams. J Manag Inf Syst.

[27] Chahine S, Cristancho S, Padgett J, Lingard L (2017). How do small groups make decisions? A theoretical framework to inform the implementation and study of clinical competency committees. Perspect Med Educ.

[28] Mohammed S, Dumville BC (2001). Team mentals models in a team knowledge framework: expanding theory and measurement across disciplinary boundaries. J Organ Behav.

[29] Wegner DM (1995). A computer network model of human transactive memory. Soc Cogn.

[30] Peltokorpi V (2008). Transactive memory systems. Rev Gen Psychol.

[31] Maynard MT, Gilson LL (2014). The role of shared mental model development in understanding virtual team effectiveness. Group Org Manag.

[32] Alavi M, Tiwana A (2002). Knowledge integration in virtual teams: the potential role of KMS. J Assoc Inf Sci Tech.

[33] Hollingshead AB, Mcgrath JE, O’Connor KM (1993). Group task performance and communication technology: a longitudinal study of computer-mediated versus face-to-face work groups. Small Group Res.

[34] Huber GP, Lewis K (2010). Cross-understanding: implications for group cognition and performance. Acad Manage Rev.

[35] De Jong BA, Dirks KT, Gillespie N (2016). Trust and team performance: a meta-analysis of main effects, moderators, and covariates. J Appl Psychol.

[36] Hambley LA, O’Neill TA, Kline TJB (2007). Virtual team leadership: the effects of leadership style and communication medium on team interaction styles and outcomes. Organ Behav Hum Decis Process.

[37] Jehn KA, Mannix EA (2001). The dynamic nature of conflict: a longitudinal study of intragroup conflict and group performance. Acad Manage J.

[38] Bell BS, Kozlowski SWJ (2002). A typology of virtual teams: implications for effective leadership. Group Org Manag.

[39] Martinez-Moreno E, Zornoza A, Orengo V, Thompson LF (2015). The effects of team self-guided training on conflict management in virtual teams. Group Decis Negot.

[40] Hemmer PA, Kelly WF (2017). We need to talk: clinical competency committees in the key of c(onversation). Perspect Med Educ.

[41] Orlitzky M, Hirokawa RY (2001). To err is human, to correct for it divine. Small Group Res.

[42] Lunenburg FC (2012). Devil’s advocacy and dialectical inquiry: antidotes to groupthink. Int J Sch Acad Intellect Divers.

[43] Swaab RI, Galinsky AD, Medvec V, Diermeier DA (2012). The communication orientation model: explaining the diverse effects of sight, sound, and synchronicity on negotiation and group decision-making outcomes. Pers Soc Psychol Rev.

[44] Nonaka I, Takeuchi H (1995). The knowledge creating company: how Japanese companies create the dynamics of innovation.

[45] Kennedy DM, Vozdolska RR, McComb SA (2010). Team decision making in computer-supported cooperative work: how initial computer-mediated or face-to-face meetings set the stage for later outcomes. Decis Sci.

[46] Kanawattanachai P, Yoo Y (2007). The impact of knowledge coordination on virtual team performance over time. Manag Inf Syst Q.

[47] Hill NS, Bartol KM, Tesluk PE, Langa GA (2009). Organizational context and face-to-face interaction: influences on the development of trust and collaborative behaviors in computer-mediated groups. Organ Behav Hum Decis Process.

[48] Majchrzak A, Malhotra A, Stamps J, Lipnack J (2004). Can absence make a team grow stronger?. Harv Bus Rev.

[49] Paul S, Samarah IM, Seetharaman P, Mykytyn PP (2004). An empirical investigation of collaborative conflict management style in group support system-based global virtual teams. J Manag Inf Syst.

[50] Paul S, Seetharaman P, Samarah I, Mykytyn PP (2004). Impact of heterogeneity and collaborative conflict management style on the performance of synchronous global virtual teams. Inf. Manage..

[51] Montoya-Weiss MM, Massey AP, Song M (2001). Getting it together: temporal coordination and conflict management in global virtual teams. Acad Manage J.

[52] O’Neill TA, Hoffart GC, McLarnon MMJW (2017). Constructive controversy and reflexivity training promotes effective conflict profiles and team functioning in student learning teams. Acad Manag Learn Edu.

[53] Lowry PB, Nunamaker JF, Curtis A, Lowry MR (2005). The impact of process structure on novice, virtual collaborative writing teams. IEEE Pro Comm.

[54] Ellwart T, Happ C, Gurtner A, Rack O (2015). Managing information overload in virtual teams: effects of a structured online team adaptation on cognition and performance. Eur J Work Organ Psychol.

[55] Penarroja V, Orengo V, Zornoza A, Sanchez J, Ripoll P (2015). How team feedback and team trust influence information processing and learning in virtual teams: a moderated mediation model. Comput Human Behav.

[56] Konradt U, Schippers MC, Garbers Y, Steenfatt C (2015). Effects of guided reflexivity and team feedback on team performance improvement: the role of team regulatory processes and cognitive emergent states. Eur J Work Organ Psychol.

[57] O’Neill TA, Lewis RJ, Hambley LA, Beyerlein S, Bradley L, Beyerlein MM, Nemiro J (2008). Leading virtual teams: potential problems and simple solutions. The handbook of high performance virtual teams: a toolkit for collaborating across boundaries.

[58] Varty CT, O’Neill TA, Hambley LA, Blount Y, Gloet M (2017). Leading anywhere workers: a scientific and practical framework. Anywhere working and the new era of telecommuting.

[59] Hambley LA, O’Neill TA, Kline TJB (2007). Virtual team leadership: perspectives from the field. Int J E Collab.

[60] Muethel M, Hoegl M, Cattani G, Ferriani S, Frederiksen L, Taube F (2011). Shared leadership functions in geographically dispersed project teams. Project-based organizing and strategic management. Advances in strategic management.

[61] Drescher G, Garbers Y (2016). Shared leadership and commonality: a policy-capturing study. Leaders Q.

[62] Hoch JE, Kozlowski SWJ (2014). Leading virtual teams: hierarchical leadership, structural supports, and shared team leadership. J Appl Psychol.

[63] Hoegl M, Muethel M (2016). Enabling shared leadership in virtual project teams: a practitioners’ guide. Proj Manag J.

[64] Johnson SD, Suriya C, Yoon SW, Berrett JV, La Fleur J (2002). Team development and group processes of virtual learning teams. Comput Educ.

[65] Tuckman BW (1965). Developmental sequence in small groups. Psychol Bull.

[66] Mittleman DD, Briggs RO, Nunamaker JF (2000). Best practices in facilitating virtual meetings: some notes from initial experiences. Group Facil.

[67] Clawson VK, Bostrom RP, Anson R (1993). The role of the facilitator in computer-supported meetings. Small Group Res.

[68] Friedman KA, Raimo J, Spielmann K, Chaudry S (2016). Resident dashboards: helping your clinical competency committee visualize trainees’ key performance indicators. Med Educ Online.

